# The accuracy of self-report versus objective assessment for estimating socioeconomic inequalities in disease prevalence in Indonesia

**DOI:** 10.1007/s00038-019-01301-5

**Published:** 2019-09-17

**Authors:** Joko Mulyanto, Dionne S. Kringos, Anton E. Kunst

**Affiliations:** 1 Department of Public Health and Community Medicine, Faculty of Medicine, Universitas Jenderal Soedirman, Purwokerto, Indonesia; 2 Department of Public Health, Amsterdam UMC, University of Amsterdam, Amsterdam Public Health Research Institute, Amsterdam, The Netherlands

**Keywords:** Socioeconomic, Inequality, Self-reported health condition, Objective assessment, Hypertension, Asthma

## Abstract

**Objectives:**

To estimate socioeconomic inequalities in hypertension and asthma prevalence in Indonesia, to compare estimates based on self-report (SR) to those based on objective assessment (OA), and to assess the role of sensitivity and specificity of SR.

**Methods:**

We used data from the 2014 Indonesia Family Life Survey (*n* = 34,257). We measured inequalities in hypertension and asthma prevalence in relation to educational level and income, using standardised prevalence rate and the relative index of inequality (RII). Using OA as standard, we calculated the sensitivity and specificity of SR.

**Results:**

For hypertension, reversed inequalities were found when estimated by SR instead of OA (RII for education 0.86, 95% CI 0.74–0.99 vs. RII 1.29, 95% CI 1.16–1.44). For asthma, a similar but even larger reversal of inequalities was found. The sensitivity of SR was low overall, and especially for the lowest education or income group.

**Conclusions:**

Results imply that the use of SR may lead to underestimation of socioeconomic inequalities in disease prevalence in a low-income country such as Indonesia. The use of OA is recommended for monitoring inequalities in non-communicable disease prevalence.

## Introduction

A reduction in health inequalities is a priority worldwide. There is considerable evidence that inequalities in disease prevalence lead to unnecessary and avoidable mortality and other negative health outcomes (Mackenbach et al. 2000, 2008). Accurate estimation of inequalities in disease prevalence is essential to identify existing and emerging inequalities that require policy action. This is particularly relevant in the context of low- and middle-income countries (LMICs), where sizeable inequalities in health are likely to exist (Hosseinpoor et al. 2012). However, accurate estimation of inequalities requires valid data on disease prevalence (Hosseinpoor et al. 2015; WHO 2013). In LMICs, studies on inequalities in disease prevalence often rely on self-reported data due to lack of objective data based on physical measurement (hereafter referred to as ‘objective assessment’). However, evidence on the accuracy of using patient-reported data on the prevalence of diseases initially diagnosed by a physician (hereafter referred to as ‘self-report’) to estimate inequalities in disease prevalence is inconclusive.

Hypertension is a disease with a high global burden and also a major risk factor for cardiovascular disorders (Kearney et al. 2005). Studies on socioeconomic inequalities in the prevalence of hypertension show inconsistent findings due to the different measurement methods applied. An international comparative study showed that the prevalence of hypertension in LMICs is consistently higher in the lowest income group compared to the highest income group when measured using objective assessment (Palafox et al. 2016). Other studies in both high-income countries and LMICs showed that, compared to objective assessment, self-report provides different estimates of socioeconomic inequalities in the prevalence of hypertension (Beltrán-Sánchez and Andrade 2016; Dalstra et al. 2005; Johnston et al. 2009; Kaplan et al. 2010; Vellakkal et al. 2013, 2014).

Although asthma is another non-communicable disease with a significant global burden (Masoli et al. 2004), few studies have examined inequalities in the prevalence of asthma. Studies using pooled data from 41 LMICs showed that socioeconomic status (SES) was inversely associated with the prevalence of asthma when measured using objective assessment (Hosseinpoor et al. 2012). A comparative study using data from six countries showed that in five countries (China, Ghana, India, Mexico and South Africa) the estimates of socioeconomic inequalities in asthma prevalence were smaller and in reverse direction when measured using self-report compared to objective assessment (Vellakkal et al. 2014).

These studies give the impression that the use of subjective indicators, e.g. the prevalence of self-report of diseases, could be prone to bias (Idler and Benyamini 1997; Jürges 2007). However, the evidence on the validity of self-report is inconclusive and mostly comes from a few high-income countries, whereas the evidence from LMICs is limited. In addition, it is unclear why the estimates of socioeconomic inequalities in disease prevalence measured by self-report may differ from inequality estimates measured by objective assessment. This issue has not been investigated in the previous studies, particularly in the context of LMICs. A first step in this understanding is to assess the sensitivity and specificity of self-report as a tool to measure disease prevalence.

The Indonesian situation allows us to investigate in more detail in an LMIC setting the use of self-report compared with objective assessment to estimate socioeconomic inequalities in disease prevalence. For such an analysis, data were available from a nationally representative survey in Indonesia which measured hypertension and asthma using patient reports of diseases initially diagnosed by a physician (self-report) and objective assessment based on physical measurements from the same individual. Previous studies on socioeconomic inequalities in hypertension prevalence in Indonesia measured by self-report showed inconsistent results (Christiani et al. 2015; Ng et al. 2006). For asthma, a report from the Indonesian Basic Health Research Study (2013) showed that the prevalence of asthma measured by self-report was higher in high-income groups (NIHRD 2013). However, these earlier studies made no comparison between disease prevalence calculated by self-report and by objective assessment.

In Indonesia, it is unknown whether estimates of socioeconomic inequalities measured by self-report differ from the objective assessment. Therefore, this study assesses the accuracy of using self-report to estimate socioeconomic inequalities in disease prevalence in Indonesia compared to using objective assessment based on physical measurement. This study also examined whether the sensitivity and specificity of self-report contribute to differences between those estimates of inequalities.

## Methods

### Study design and population

This cross-sectional study used data from the 5th wave of the Indonesia Family Life Survey (IFLS5) conducted in 2014 by the RAND Corporation (USA). The IFLS5 was approved by the relevant ethical review committees in the USA and Indonesia. The data are publicly accessible through RAND’s website, and more details on the IFLS are published elsewhere (Strauss et al. 2016).

The IFLS collected data from 13 Indonesian provinces that, together, represent 83% of the Indonesian population. The present study sample consisted of 34,257 IFLS5 participants aged ≥ 15 years who had complete data on all study variables and received no medication for related diseases. After data cleaning, 31,247 (91.3%) and 32,267 (94.1%) persons were included in the analysis for hypertension and asthma, respectively. A detailed description of individuals included in the analysis, which excluded those on relevant medication, is provided in Table 1.

**Table 1:** Number of respondents in the analysis (Indonesia, 2014)

	Hypertension	Asthma
Eligible respondents	34,257	34,257
Complete data on self-report and objective assessment	32,243	32,423
Complete data on self-report, objective assessment, and demographic details and SES^a^	32,067	32,267
Complete data on self-report, objective assessment, demographic details and SES, excluding respondents on medication	31,247	32,267
Percentage of respondents included in analysis (of total eligible respondents)	91.3	94.1

^a^Socioeconomic status

### Variables and outcomes measured

SES was estimated based on educational level and income. Education level was defined according to the 2011 International Standard Classification of Education (UNESCO-UIS 2012). Based on the highest level completed by each individual, education level was categorised into five groups (pre-primary, primary, lower secondary, upper secondary and tertiary level).

The level of household consumption was used as a proxy for income. In developing countries, consumption is considered the most valid direct measurement of income or household wealth (O’Donnel et al. 2008). Household consumption, including non-food items, is chosen as it reflects the actual level of household resources, including resources obtained from the non-market transaction and home production, which is common in LMIC’s setting such as Indonesia. We did not measure gained income as many people work in the informal sector, have multiple and continually changing sources of income, or live on home production. As a result, gained income tends to be received intermittently and from various sources, making it difficult to obtain valid estimates of monetary income. The household consumption counted food, non-food consumables, durable goods, spending on education and housing. These counts were aggregated and adjusted into a monthly estimate, which was adjusted to household size to account for economics of scale. We adjusted for geographical differences in purchasing power parity between provinces and between urban and rural areas, by rescaling respondents’ income according to poverty line corresponding to their province and urban versus rural location of residence. Information on poverty line was obtained from the Indonesian Central Bureau of Statistics. For further analysis, the household’s income was grouped into quintiles.

The prevalence of hypertension and asthma was measured using self-reported morbidity and objective assessment. Self-reported data were obtained from the IFLS5 based on response to the question: *Has a doctor/paramedic/nurse/midwife told you that you have hypertension or asthma?* The IFLS5 collected data of standard blood pressure measurements, which were in line with the American Heart Association standard [35]. Blood measurement data from the IFLS were used to identify a diagnosis of hypertension based on the 7th Joint National Committee classification (James et al. 2014). The mean systolic/diastolic blood pressure was calculated for three consecutive measurements; a mean systolic blood pressure ≥ 140 mmHg and/or a diastolic blood pressure ≥ 90 mmHg was classified as hypertension.

To establish the diagnosis of asthma, peak expiratory flow (PEF) data were obtained from the IFLS5; the highest value of three PEF measurements was used. A diagnosis of asthma was established when a respondent’s PEF was ≤ 50% of the predicted PEF value that was predicted based on age, gender and height (Radeos and Camargo 2004). Numbers of respondents living in urban versus rural locations and the provinces (Java/Bali and others) were obtained from the IFLS5.

### Statistical analysis

The following data were calculated: overall prevalence rates for hypertension and asthma, as well as prevalence rates by educational level, income quintile and geographical area. Prevalence rates were calculated based on self-report and objective assessment. Prevalence rates were measured as number of cases per 100 persons; these rates were age- and sex-standardised using the direct method, with the total survey population as the population. Then, the difference was calculated between the standardised prevalence rate of self-report and of objective assessment for each population group; this was calculated by subtracting the prevalence rate of diseases measured by objective assessment from the self-report, for each population group (Fleiss et al. 2003).

The relative index of inequality (RII) was used to estimate the magnitude of socioeconomic inequalities in disease prevalence in a more comprehensive way. The RII is a regression-based index that assesses the probability of disease in relationship to the relative hierarchical position of every individual within the socioeconomic hierarchy. A higher RII indicates a stronger association between this hierarchical position and disease prevalence. This implies a greater disease prevalence difference between the lower and higher SES groups: RII = 1 indicates equality, RII < 1 indicates negative inequality with higher prevalence rates among higher SES, and RII > 1 indicates positive inequality with higher prevalence among lower SES. The RII is a valid health inequality measure to facilitate comparisons across diverse populations and outcomes (Mackenbach and Kunst 1997). The regression model was adjusted for age and sex. The value of the RII (95% CI) for the prevalence of hypertension and asthma was compared between self-report and objective assessment. For geographical characteristics, the odds ratio (OR; 95% CI) of the prevalence of hypertension and asthma was calculated using logistic regression; then, the ORs of the self-report and objective assessment were compared.

Sensitivity was defined as the prevalence in respondents who had (self-reported) hypertension/asthma and in those who had hypertension/asthma based on objective assessment. Specificity was defined as the prevalence of respondents who (self-reported) to have no hypertension/asthma and those who had no hypertension/asthma based on objective assessment. The sensitivity and specificity were stratified by SES and geographical characteristics.

## Results

### Sample characteristics

In this study population, the characteristics of the respondents with hypertension and asthma were largely similar regarding sex, age, education level and geographical location (Table 2).

**Table 2:** Characteristics of the study population by diseases (Indonesia, 2014)

	Hypertension		Asthma	
	*N*	%	*n*	%
Sex				
Male	14,761	47.2	15,097	46.8
Female	16,486	52.8	17,170	53.2
Age group (years)				
15–29	10,689	34.2	10,761	33.3
30–39	8350	26.7	8489	26.3
40–49	5418	17.3	5623	17.4
50–59	3560	11.4	3844	11.9
60–69	1899	6.1	2104	6.5
≥ 70	1331	4.3	1446	4.5
Education level				
Pre-primary	6041	19.3	6361	19.7
Primary	6780	21.7	7003	21.7
Lower secondary	6644	21.3	6765	21.0
Upper secondary	8954	28.7	9176	28.4
Tertiary	2828	9.1	2962	9.2
Income^a^				
1st quintiles (162–1300)	6248	20	6453	20
2nd quintiles (1300–1830)	6249	20	6453	20
3rd quintiles (1830–2500)	6251	20	6454	20
4th quintiles (2500–3790)	6249	20	6454	20
5th quintiles (3790–4,570,000)	6250	20	6453	20
Location				
Urban	18,274	58.5	18,951	58.7
Rural	12,973	41.5	13,316	41.3
Province				
Java and Bali	18,558	59.4	19,200	59.5
Others	12,689	40.6	13,067	40.5

^a^Range of income in each quintile in thousands Indonesian Rupiah (IDR)

### Socioeconomic inequalities in hypertension and asthma

Table 3 presents the estimates of socioeconomic inequalities in the prevalence of hypertension/asthma by objective assessment and self-report. Overall, when estimated by self-report, the prevalence of hypertension was 10.14 per 100 persons compared to 22.25 per 100 persons with objective assessment. For the different SES groups, the prevalence rates of hypertension were much lower when estimated by self-reporting diagnosis compared to objective assessment. The largest differences in the hypertension prevalence rate between self-report and objective assessment were in the lowest SES groups (13.4 per 100 persons). For both educational level and income, the prevalence rates of hypertension measured by self-report were lower in the lowest SES group compared to the highest SES group, whereas the prevalence rates measured by objective assessment were always higher in the lowest SES groups compared to the highest SES groups. Using RII confirmed substantial differences in the estimate of socioeconomic inequalities in hypertension prevalence when estimated by self-report compared to objective assessment. Educational inequalities in the prevalence of hypertension showed RII 0.86 (95% CI 0.74–0.99) when estimated by self-report compared to RII 1.29 (95% CI 1.16–1.44) with objective assessment.

**Table 3:** Estimates of socioeconomic inequalities in prevalence of hypertension and asthma by objective assessment and self-report (Indonesia, 2014)

	Hypertension			Asthma		
	Objective assessment	Self-report	Difference	Objective assessment	Self-report	Difference
*Overall prevalence* ^*a*^	22.25	10.14	12.11	5.03	2.94	2.09
Education level						
SPR^b^						
Pre-primary	23.23	9.83	13.40	6.04	2.94	3.10
Primary	22.76	10.15	12.61	5.49	2.45	3.04
Lower secondary	20.17	9.44	10.73	4.57	3.04	1.53
Upper secondary	21.90	11.11	10.79	3.27	2.98	0.29
Tertiary	21.29	10.07	11.22	3.37	3.74	− 0.37
RII^c^ (95% CI)	1.29 (1.16–1.44)	0.86 (0.74–0.99)	–	3.05 (2.46–3.78)	0.82 (0.65–1.04)	–
Income						
SPR^b^						
1st quintiles	22.97	9.52	13.45	6.06	2.66	3.40
2nd quintiles	21.72	9.92	11.80	5.17	2.37	2.80
3rd quintiles	21.99	10.00	11.99	4.83	2.86	1.97
4th quintiles	22.08	10.72	11.36	4.68	2.91	1.77
5th quintiles	22.41	10.65	11.76	3.87	3.93	− 0.06
RII^c^ (95% CI)	1.02 (0.92–1.12)	0.78 (0.69–0.90)	–	2.1 (1.77–2.54)	0.61 (0.49–0.77)	–
Location						
SPR^b^						
Urban	22.64	10.45	12.19	4.73	3.13	1.60
Rural	21.75	9.71	12.04	5.40	2.67	2.73
OR^d^ (95% CI)	1.08 (1.02–1.15)	1.11 (1.03–1.20)	–	0.84 (0.75–0.93)	1.16 (1.01–1.32)	–
Province						
SPR^b^						
Java and Bali	22.21	9.99	12.22	5.43	3.07	2.36
Others	22.32	10.37	11.95	4.06	2.76	1.30
OR^d^ (95% CI)	0.99 (0.94–1.05)	0.96 (0.89–1.04)	–	0.81 (0.73–0.91)	0.90 (0.78–1.02)	–

^a^Per 100 persons; ^b^*SPR* standardised prevalence rate, directly standardised to age and sex per 100 persons; ^c^*RII* relative index of inequality; *CI* confidence interval; ^d^*OR* odds ratio

Similar findings emerged for the prevalence of asthma. The overall prevalence of asthma was 2.94 per 100 persons when estimated by self-report compared to 5.03 per 100 persons with objective assessment. The largest differences in the prevalence of asthma, when estimated by self-report compared to objective assessment, was found in the lowest SES groups (about 3 per 100 persons). Different socioeconomic gradients in the prevalence of asthma were observed between self-report and objective assessment. Also, the largest difference in educational inequalities in the prevalence of asthma showed RII 0.82 (95% CI 0.65–1.04) when estimated by self-report compared to RII 3.05 (95% CI 2.46–3.78) with objective assessment.

In urban versus rural areas, and the provinces, the prevalence of hypertension and asthma was substantially smaller when measured by self-report compared to objective assessment. However, the difference in the prevalence rate of hypertension was similar between the different areas and provinces (about 12 per 100 persons). For asthma, larger differences in the prevalence rate between self-report and objective assessment were found in rural areas (2.73 per 100 persons) and the provinces of Java and Bali (2.36 per 100 persons). In most geographical areas, similar ORs were found for the prevalence of hypertension and asthma when measured by self-report compared to objective assessment, except for ORs of asthma prevalence between urban versus rural areas, i.e. OR 1.16 (95% CI 1.01–1.32) measured by self-report compared to OR 0.84 (95% CI 0.75–0.93) measured by objective assessment.

### Sensitivity and the specificity of self-report

Sensitivity and specificity of self-report were analysed using objective assessment as the gold standard and then stratified by SES groups and geographical characteristics (Table 4). Overall, the sensitivity of self-report was very low for the prevalence of hypertension (26.56%) and even lower for asthma (11.95%). However, there was a high specificity of self-report for the prevalence of hypertension (94.57%) and asthma (97.54%). The sensitivity of self-report for the prevalence of hypertension was lowest in the lowest SES groups and gradually increased in the higher SES group. Lower sensitivity of self-report was found in low SES groups for the prevalence of asthma compared to the high SES groups. However, the sensitivity did not increase linearly from low SES groups to higher SES groups in asthma prevalence. A similar high specificity was found among the SES groups in the prevalence of both hypertension and asthma, except for the specificity of asthma prevalence, which was slightly lower in the highest SES group. This implies that some individuals in the highest SES group reported having asthma, although this was not diagnosed using objective assessment.

**Table 4:** Sensitivity and specificity of self-report for hypertension and asthma prevalence by socioeconomic status and geographical characteristics (Indonesia, 2014)

	Hypertension		Asthma	
	Sensitivity	Specificity	Sensitivity	Specificity
*Overall*	26.56	94.57	11.95	97.54
Education level				
Pre-primary	24.31	94.59	11.24	97.65
Primary	25.75	94.51	10.01	98.01
Lower secondary	28.25	95.27	13.56	97.46
Upper secondary	28.78	93.99	16.87	97.49
Tertiary	29.28	94.84	13.49	96.63
Income				
1st quintiles	24.50	95.15	10.21	97.98
2nd quintiles	25.69	94.30	12.33	98.06
3rd quintiles	26.00	94.76	12.19	97.62
4th quintiles	27.41	94.09	13.57	97.54
5th quintiles	29.11	94.56	15.47	96.50
Location				
Urban	27.54	94.52	12.34	97.33
Rural	25.22	94.64	11.54	97.84
Provinces				
Java and Bali	27.01	94.87	12.08	97.45
Others	25.87	94.11	11.71	97.66

## Discussion

### Key findings

This study, conducted in Indonesia, shows no (or relatively small) socioeconomic inequalities in the prevalence of hypertension and asthma when measured by self-report. These findings are in accordance with earlier results from the 2013 Indonesian Basic Health Research Study, which also used self-report (NIHRD 2013). However, relatively large socioeconomic inequalities in hypertension and asthma prevalence were found when measured by objective assessment. Moreover, the direction of the inequalities by both education and income level changed when measuring inequalities based on objective assessment as opposed to self-reported diagnoses. These findings indicate that the differences in estimates of inequality in disease prevalence based on the different measurement methods might be attributed to the low sensitivity of self-report, particularly in low SES groups.

### Interpretation

Findings from our study are in line with a recent report from the WHO, which estimated the prevalence of hypertension using objective assessment (Hosseinpoor et al. 2017). Our results are also consistent with two previous studies in LMICs, which compared self-report with objective assessment to estimate socioeconomic inequalities in disease prevalence. Use of self-report provided higher relative rates of disease prevalence in high SES groups, while higher relative rates of disease prevalence were found in low SES groups when measured by objective assessment (Vellakkal et al. 2013, 2014). An international comparative study showed that these differences were even larger in low-income countries than in middle-income countries (Vellakkal et al. 2014).

Our findings show that using self-report leads to an underestimation of disease prevalence and socioeconomic inequalities; this is probably associated with the low sensitivity of self-report across all SES groups and, particularly, in low SES group. The low sensitivity of self-report may have various causes. First, since these diseases can be asymptomatic, those who are asymptomatic may not seek medical care. Hypertension is a non-communicable disease that is asymptomatic or does not have specific symptoms. In Indonesia, most individuals, particularly in low SES groups, do not have a routine medical checkup and seek medical care only when they have severe symptoms (Hussain et al. 2016; Setiati and Sutrisna 2005). Also, there is no systematic and nationwide screening programme for hypertension. All this may contribute to the underreporting of hypertension in all SES groups. However, this assumption does not apply for asthma, which is a chronic disease with clear symptoms.

A second possibility is that individuals have symptoms but may lack access to healthcare facilities. To be diagnosed with a disease, the individual must have access to healthcare and be seen by trained persons. Low or lack of access to healthcare contributes to underreporting of diseases. A study in Indonesia showed that low utilisation of healthcare was associated with a higher probability to have uncontrolled hypertension (Hussain et al. 2016). Studies from China and Thailand also support our idea that low reporting of chronic conditions in low SES groups is probably influenced by limited access to healthcare (Zimmer and Amornsirisomboon 2001; Zimmer and Kwong 2004).

Third, in Indonesia, individuals diagnosed in a healthcare facility may not necessarily be told that they have the disease. In Indonesia, doctor–patient communication is characterised by a hierarchical relationship and is, generally, a one-way method of communication (Claramita et al. 2011, 2013). An Indonesian study found that a lack of good communication between physician and patient generally leads to poor information about having a disease, particularly in low SES groups (Sari et al. 2016). Previous systematic reviews concluded that patients with low SES tend to receive less information from physicians about their health conditions, e.g. information on disease diagnosis and treatment. This can be due to factors on both sides, e.g. patients from low SES groups have a less active communication style and elicit less response from physicians; on the other hand, physicians may inaccurately assume that patients from low SES groups are not interested in learning about their health or will not understand the information (Verlinde et al. 2012; Willems et al. 2005).

Fourth, they may have been informed that they are diagnosed with a disease but may not understand this explanation. Lack of knowledge about the disease will be a barrier to reporting having the disease, even when they have been informed about the disease. Health literacy is likely to play a major role in this underreporting. Although no studies in Indonesia have specifically investigated the association between health literacy and reporting of chronic disease, some Indonesian studies showed low educational level to be associated with low awareness of hypertension (Christiani et al. 2015; Hussain et al. 2016). Studies in high-income countries show that health literacy plays a major role in chronic disease outcome, due to low knowledge of the diseases. People with low education show less active health information-seeking behaviour, which often leads to late recognition of a chronic health condition (Beacom and Newman 2010; Gazmararian et al. 2003). This explanation is supported by our finding that the socioeconomic gradient in the sensitivity of self-report is more strongly observed in the education level than in the income level for both hypertension and asthma.

A final explanation for the low sensitivity of self-report is that patients may be aware that they have been diagnosed, have been informed and understood the explanation, but may not report this when asked in a survey (Johnston et al. 2009). Several factors may contribute to this underreporting. We argue that cultural factors in different geographical areas lead to different behaviours in the self-reporting of diseases. In most eastern countries such Indonesia, people have a culture which maintains ‘life modesty’ as a principal value, especially when talking to other people (Kandula et al. 2007; Maty et al. 2011). It is considered impolite and ungrateful to complain about problems in their life, including health issues; this factor is probably stronger among people with lower SES and living in rural areas. People in high SES groups tend to adhere less to traditional values, are more adapted to Western values, more straightforward, have higher expectations and find it more acceptable to complain about health-related problems. A study in Indonesia found that people with high SES or live in urban areas more often reported risk factors for non-communicable diseases when interviewed in a health survey (Ng et al. 2006). This is also consistent with the fact that, in the present study, the sensitivity of self-report is lower in rural areas.

### Strengths and limitations

A strength of the present study was the use of the IFLS5 dataset with a study sample based on the national economic survey sampling frame from the Statistics Bureau, Indonesia; this makes our findings generalisable to the Indonesian population. In addition, the objective assessment was used to meet global standards in disease measurement, making these results suitable for international comparisons.

Nevertheless, some limitations also need to be addressed. First, from the various groups, 9% and 6%, respectively, of eligible respondents were excluded from the analysis; since these excluded persons were not randomly distributed between the different demographics and SES groups, this may influence the estimate of socioeconomic inequalities. Second, we cannot fully eliminate the possibility of measurement bias in the objective assessment based on physical measurement; although both the measurement instrument and the technique were standardised, the large-scale and repeated measurements, and the various geographical areas which had to be covered could have caused measurement bias. Third, our estimation of the prevalence of asthma was based on PEF measurement, which is not the gold standard (i.e. spirometry measurement) to establish an asthma diagnosis in a clinical setting. However, in many large-scale health surveys and population-based studies, PEF is still widely used because the validity and reliability of the measurement are adequate, and it is less costly and more practical than spirometry measurement. Nevertheless, there is a possibility of measurement bias.

### Conclusions

This study shows that estimates of socioeconomic inequalities in the prevalence of hypertension and asthma using self-report based on a single question were smaller and showed a reversed direction of inequalities. This indicates that the results of studies using self-report to measure disease prevalence should be interpreted with caution. To measure disease prevalence, such as the burden of disease and health inequalities, we recommend using objective methods based on physical assessment. If this is not feasible, studies in other LMICs with a similar setting have shown that the use of a multiple-question instrument, based on disease symptoms, is likely to improve the accuracy of such an estimation.

The underreporting of non-communicable diseases is indicative of a more generalised problem in the access, utilisation and quality of healthcare in Indonesia by the poor or those with lower education. The failure of these people to identify and report their disease calls for measures such as developing more proactive preventive care services, improving the physician–patient relationship and communication and increasing the health literacy of the people with lower education. Further studies should look into the role of specific factors, e.g. those related to poor access to high-quality healthcare services, in order to understand the low sensitivity of self-report in LMICs such as Indonesia, particularly for the lowest SES groups.
